# A large-scale application of project prioritization to threatened species investment by a government agency

**DOI:** 10.1371/journal.pone.0201413

**Published:** 2018-08-14

**Authors:** James Brazill-Boast, Moira Williams, Beth Rickwood, Thalie Partridge, Grant Bywater, Bronwyn Cumbo, Ian Shannon, William J. M. Probert, Julie Ravallion, Hugh Possingham, Richard F. Maloney

**Affiliations:** 1 Office of Environment and Heritage, New South Wales Government, Sydney, NSW, Australia; 2 Centre of Excellence for Environmental Decisions, University of Queensland, Brisbane, Australia; 3 Department of Conservation, Christchurch, New Zealand; University of Sydney, AUSTRALIA

## Abstract

In a global environment of increasing species extinctions and decreasing availability of funds with which to combat the causes of biodiversity loss, maximising the efficiency of conservation efforts is crucial. The only way to ensure maximum return on conservation investment is to incorporate the cost, benefit and likelihood of success of conservation actions into decision-making in a systematic and objective way. Here we report on the application of a Project Prioritization Protocol (PPP), first implemented by the New Zealand Government, to target and prioritize investment in threatened species in New South Wales, Australia, under the state’s new *Saving our Species* program. Detailed management prescriptions for 368 threatened species were developed via an expert elicitation process, and were then prioritized using quantitative data on benefit, likelihood of success and implementation cost, and a simple cost-efficiency equation. We discuss the outcomes that have been realized even in the early stages of the program; including the efficient development of planning resources made available to all potential threatened species investors and the demonstration of a transparent and objective approach to threatened species management that will significantly increase the probability of meeting an objective to secure the greatest number of threatened species from extinction.

## Introduction

It has become increasingly obvious to government agencies and organisations responsible for managing and conserving biodiversity, that the resources required to adequately prevent species decline are far outweighed by those available [1–2]. Over the past several years this problem and its potential solutions has received increasing focus in the scientific literature; in particular, methods for objectively and efficiently allocating resources that draw on triage theory, cost-effectiveness analysis and decision theory [3–8]. Such methods build on the framework of ‘systematic conservation planning’ [9] which is used—either explicitly or implicitly–by many conservation practitioners and can readily be incorporated into existing decision making processes. Despite this, government agencies in general have been slow to adopt these solutions explicitly. A notable exception is the New Zealand Department of Conservation (DOC), which has implemented a cost-effective prioritization of investment in threatened species management [6,10–11].

As of August 2017 there were 917 species listed as *Vulnerable*, *Endangered* or *Critically endangered* under the *Biodiversity Conservation Act* (2016) (BC Act) in New South Wales (NSW). The NSW Office of Environment and Heritage (OEH) has a statutory responsibility to prevent the extinction and promote the recovery of all species listed under the Act. A recent review [12] indicated that existing programs for recovering threatened species in NSW were insufficient to achieve this goal. This situation is not new. After 12 years of implementing the previous *Threatened Species Conservation Act* (1995) only 10% of threatened species in NSW had a Recovery Plan (statutory document detailing a species’ status and management requirements) prepared, and when a revised strategy detailed proposed management actions for all threatened species (*Threatened Species Priorities Action Statement*), less than half of the required actions (on average) for each species were implemented and only 15% of species had >80% implementation over three years [13].

This situation is not unique to NSW; only 30% of species listed under Australia’s Environment Protection and Biodiversity Conservation Act 1999 have Recovery Plans prepared [14], expenditure on recovery of species listed under the US Endangered Species Act is less than 20% of what is required [15], and only 52% of targets were met for species recovery by the UK government recently [16]. The availability of funding for implementing recovery action, however, is only part of the story. Allocating limited funds in a strategic and efficient manner is crucial to the success of programs designed to prevent species extinction.

Determining the quantity of resources made available to government agencies for recovering threatened species is a values-based socio-political decision. How to allocate those resources to ensure the best possible outcome for the largest number of species is a question that can and should be answered in an objective way [5]. The evidence suggests that historically, for many jurisdictions, decisions about where and how to spend threatened species funding have been driven primarily by non-strategic considerations (e.g. species’ charisma / popular appeal; [14,17]). Alternatively, relative investment can be predicted by species’ threat status (i.e. those with greater risk of extinction receive more funding; [18–19]), as has been the case in NSW [13]. Neither scenario is likely to result in optimal return on investment with respect to all species’ viability.

One way to ensure that return on investment in threatened species is maximised is to set clear objectives in terms of species outcomes [20] and then prioritize spending on species, locations and/or actions via a method that incorporates the relative cost, likelihood of success and predicted benefit of alternatives [5–6]. This approach has been adopted by OEH, as recommended by [21] and following the recent success of DOC in implementing a Project Prioritization Protocol (PPP; [6]). Similar to DOC’s application of a PPP, we acknowledged that the approach is not suitable for all threatened species, hence the development of six *Management Streams* used to categorise species based on their ecological and/or management requirements [22], with the PPP applied only to *site-managed* species (the largest stream, with approximately 45% of all species).

Here we describe the process of developing a prioritization for investment in threatened species management undertaken by OEH as part of a broader refinement of the agency’s threatened species program–*Saving our Species* (SoS)–launched in December 2013 (http://www.environment.nsw.gov.au/savingourspecies/about.htm). A full explanation of the management framework is outlined in, *Saving our Species Technical Report* [22]. We followed a similar process to [6]; first defining a program-level objective (from which flowed species-level objectives), then defining species management prescriptions (conservation projects), quantifying benefits, costs and likelihood of success, and finally, ranking projects based on cost-efficiency in meeting the stated objectives. Some key differences from the DOC approach included how projects were weighted (i.e. weightings for endemism and taxonomic distinctiveness were not applied), an additional step of incorporating uncertainty in priority setting, and establishing a 100 year time horizon for objectives.

## Methods

### Defining assets

Of the 917 species listed under the BC Act, only a subset was appropriate to include in a prioritization of management investment based on cost efficiency. Species fitting the following criteria were excluded from the prioritization:

1. Those with insufficient data or expert knowledge on distribution, ecology and/or management requirements available to develop an effective management project (*data-deficient management stream*);
2. those that do not currently require any active intervention or investment beyond existing policies to be secure in the long term (*keep watch management stream*);
3. those having a large geographic range and/or being highly mobile and/or highly dispersed (constrains the ability to spatially define management or objectives) (*landscape management stream*); and
4. those with less than 10% of their total population occurring within NSW (generally either common in, or management is coordinated by other jurisdictions, therefore a lower priority for investment) (*partnership management stream*).

A more detailed explanation of species’ allocation to different management streams under the SoS program can be found in the *Saving our Species Technical Report* [22].

After applying these filters, 368 species (independent assets; 312 plants, 47 animals and 9 fungi) were selected for inclusion in the prioritization process (as of December 2013; additional species have been included since). Generally, species included in the prioritization had discrete populations that could be geographically defined, critical threats at those sites that could be identified and feasibly managed (given resources), and it was predicted that mitigation of those threats at key sites would secure the species from extinction in NSW in the long term.

### Setting objectives

The overall objective of the SoS program was to maximise the number of threatened species that are secure in the wild in NSW for 100 years. Consequently, the objective for each of the 368 species selected for prioritization was to have a 95% probability of having a viable population of the species in 100 years (criterion for being *secure*) (with a secondary objective of ensuring that the species’ threat status does not decline (e.g. from ‘Endangered’ to ‘Critically endangered’).

A ‘viable population,’ in this context, was defined as a (sub)population for which: i) all deterministic threats are controlled; ii) population size is sufficient to avoid demographic problems (i.e. ≥ ‘minimum viable population size; e.g. [23–24]); iii) population trajectory is stable or increasing; and iv) there is sufficient suitable habitat for the population to persist and grow. This objective equates, effectively, to reducing a given species’ 100-year extinction risk to 5%. The aim of defining such an objective was to articulate the minimum required long-term outcome of any given species’ project, which could then drive the development of an appropriate management prescription.

### Developing projects

Each species’ conservation project comprised a suite of management and monitoring actions proposed at a set of identified sites within NSW, designed to meet the stated objective (see above). The term ‘site’ had a practical definition for the purposes of project development; referring to a spatially defined area, which encompasses one or more locations where a particular threatened species is known to occur and where any given threat to that species is managed in a consistent way.

**Fig 1:**
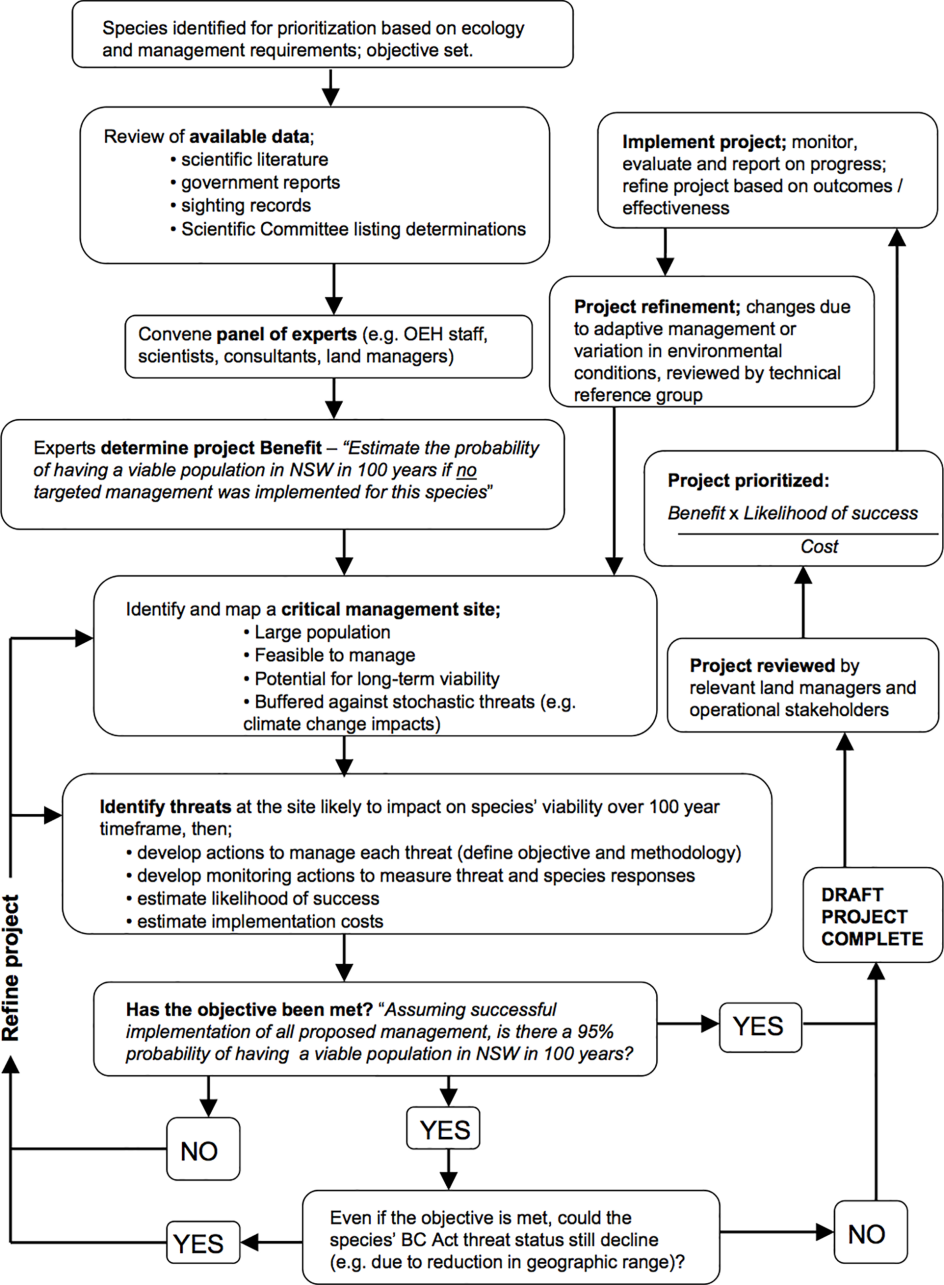
Key stages in the development and prioritization of projects; a continuous cycle with new species added as they are listed on the Schedules of the NSW Biodiversity Conservation Act (2016).

To develop each project, a structured elicitation workshop (1–3 hours duration; Fig 1) was conducted with a panel of (1–8) experts on the species ecology, distribution, threats and management requirements. Interviews were mediated by 1–2 OEH staff members, capturing both text and spatial data based on panel responses. Experts were identified via authorship of relevant publications (e.g. recovery plans, research papers) and also included individuals with relevant experience in threatened species management from state agencies, natural resource management agencies, ecological consultancies and non-government conservation organisations. Workshops were conducted over an 18-month period during 2011–2012, involving a total of 262 experts.

Prior to each workshop, a review of all available information pertaining to the species and/or its management was undertaken (e.g. peer-reviewed and ‘grey’ literature, recovery/management plans, survey reports and statutory threatened species determinations). Species varied with respect to the availability of relevant data on their ecology, demography and management requirements. Wherever possible, published data were used to inform the development of projects, however, where this was insufficient, expert opinion was relied upon.

Given the variable but finite budget for managing threatened species in NSW (similar to most jurisdictions worldwide; [2]), there is an implicit trade-off between the number of species that can be effectively managed and the quantity of resources that can be invested in each species. Therefore, the resource requirements identified for project implementation must correspond to the minimum extent required to secure the species (i.e. 95% probability of having a viable population in 100 years). The primary aim of each workshop, then, was to identify the minimum number of management sites, and actions required to abate *critical* threats at those sites. Standard monitoring actions were developed for all sites and projects, designed to track species’ populations through time (i.e. species response monitoring) and evaluate management effectiveness (i.e. threat response monitoring). To ensure that the additional (and highly variable) costs associated with monitoring did not skew the prioritization, only critical, point-of-investment monitoring activity required to inform adaptive management was included in projects.

All members of each expert panel were interviewed together in order to facilitate discussion and information exchange. Often it is recommended that expert elicitation of this type be structured (e.g. using Delphi techniques) to minimise bias and the potential for participants to influence each other’s estimates, as well as to accurately quantify confidence intervals [25–26]. The nature of much of the information being sought, however, generally precluded the use of this technique. The expertise of individual participants, particularly conservation practitioners, was often site-specific, with no single participant on the panel having expert knowledge of the species’ ecology or habitat requirements across its entire range. Therefore, when making complex decisions requiring the integration of this knowledge, such as determining the relative importance of management sites to the species’ state-wide viability, group discussion and consensus were important.

### Site selection

As a guiding principle, experts were advised to select sites in order to maximise the likelihood of meeting the project objective and minimise cost; explicitly considering population size, habitat condition, extent/severity of threatening processes and feasibility of management (e.g. managing sites on public tenure is generally more feasible than on privately owned land). Sites (and concomitant threats and actions) were identified and added to the project iteratively, until there was consensus among the panel that the project was likely to meet its objective, assuming full and successful implementation of all proposed actions (Fig 1). When determining the *number* of sites sufficient to meet the project objective, experts were guided to consider explicitly the likelihood of stochastic or unpredictable (i.e. unmanageable) threats (e.g. infectious disease, wildfire, climate change impacts) and surrogate variables known to affect extinction risk, e.g. population size, population decline, geographic range, and connectivity [27–28]. Given the long timeframe and likely effects of climate change and stochastic processes on subpopulation viability, a precautionary approach was adopted. In practice, this meant that (where known) multiple subpopulations were identified for management, in order to buffer against these types of threats.

Seventy-nine (21%) species were only known to occur in one location. Identifying only a single site for management was generally deemed inadequate for securing a species in the long term, given the risk associated with stochastic events. In such cases, actions were proposed to facilitate the establishment of additional (sub)populations via either targeted survey, translocation or ex-situ management.

### Calculating benefit

The benefit (*B*) of each project was defined as the marginal increase in the species’ probability of viability over 100 years attributable to investment in management intervention (0–1). This time horizon, although relatively long (subjecting estimates of *B* to greater uncertainty), was considered appropriate because it prompted experts to consider long-term threats–in particular climate change–when developing projects. In addition, estimates of population viability for long-lived species (and/or those with long generation times) was more meaningful over a longer timeframe. Project benefit was calculated using the formula:

$$B=P_{w}-P_{n}$$

where *P*_*w*_ is the species’ probability of having a viable population in 100 years *with* full and successful implementation of the proposed project (over the requisite timeframe), and *P*_*n*_ is the equivalent probability *without* any targeted management being implemented for the species. Given that the objective of all projects was that *P*_*w*_ ≥ 0.95 (i.e. experts were instructed specifically to design projects that were predicted to reduce 100-year extinction risk to ≤ 5%), only a relatively small number (*n =* 22) of projects (e.g. those significantly affected by unmanageable or unpredictable threats and/or having very small population with inherently high extinction risk) had *P*_*w*_ <0.95 (range: 0.5–0.9). All remaining projects were therefore allocated a default value of 0.95 for *P*_*w*_. Consequently, variation in *B* was explained primarily by variation in *P*_*n*_.

**Fig 2:**
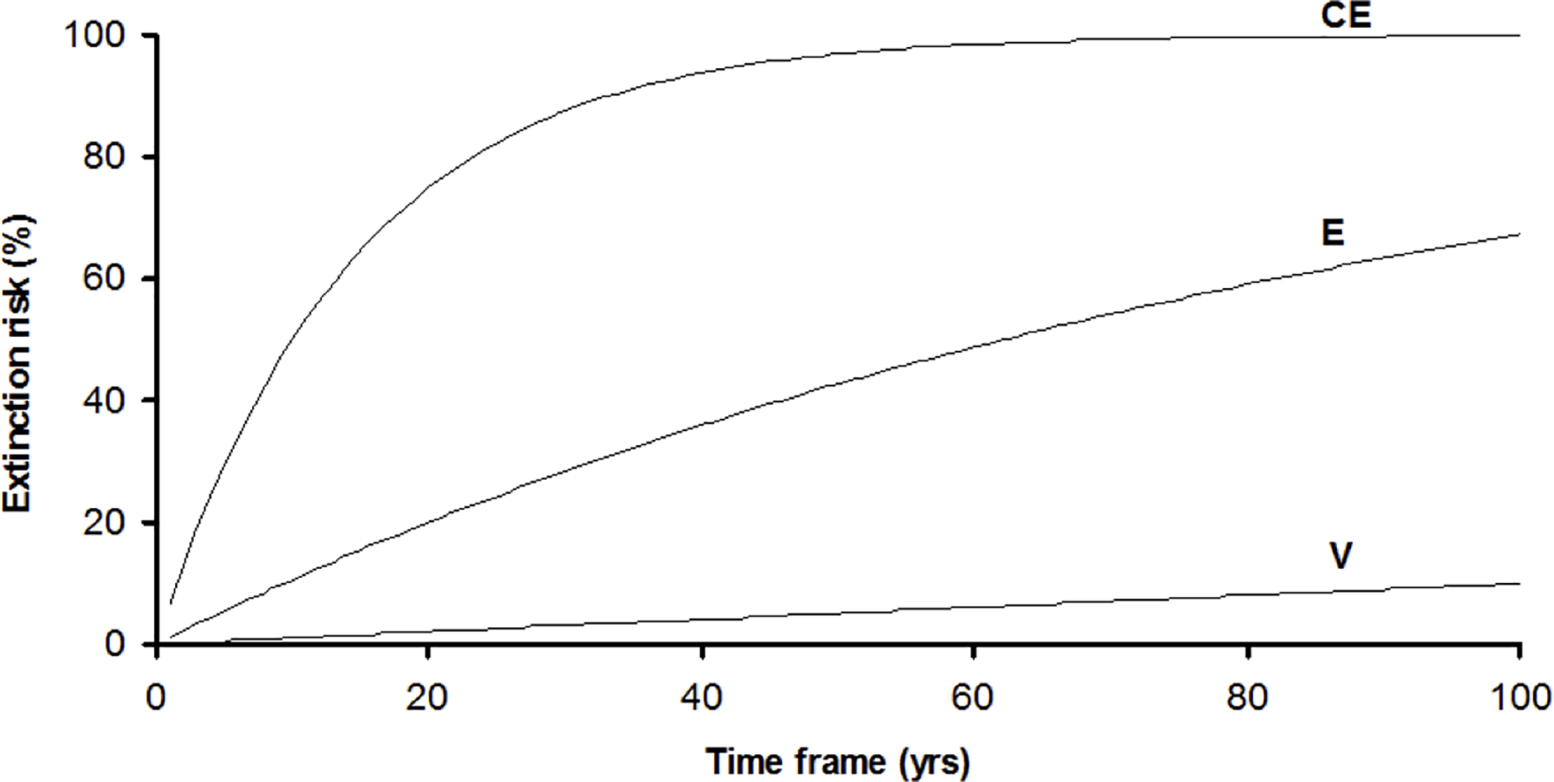
Cumulative extinction risk curves for three IUCN Red List categories. *Vulnerable* (V), *Endangered* (E), and *Critically* endangered (CE). Reproduced from Kindvall, O., and U. Gärdenfors (2003) Temporal extrapolation of PVA results in relation to the IUCN Red List criterion E. Conservation Biology **17:** 316–321.

*P*_*n*_ was elicited from the expert panel by asking the question, ‘*Assuming that the species receives no targeted management* (*beyond status quo management; i*.*e*. *a general level of protection afforded by reserve management and development regulation*), *what is the estimated probability of the species having a viable population 100 years from now*?’ This is a difficult and complex question required predicting the dynamics of threatening processes and their likely effects on populations in the future. Therefore estimates elicited from different experts were likely to be relatively uncertain and or variable between comparable species. To help reduce this uncertainty and variation, and to help guide experts, anchoring values were developed based on an IUCN Red List assessment [29] (representative of extinction risk). Each species was assessed against the criteria, where data were available (e.g. population size and decline, extent of occurrence, area of occupancy), and allocated to the appropriate threat category (*Vulnerable*, *Endangered* or *Critically endangered*). Where sufficient data were not available, the species’ equivalent BC Act threat status was used instead. This category was then converted to a 100-year extinction risk following the method proposed by [30] (Fig 2) (and supported by [31]). Based on their model (Fig 2), and assuming *P*_*n*_ to be the inverse of 100-year extinction risk, anchoring values for *P*_*n*_ applied to Vulnerable, Endangered and Critically endangered species were 0.9, 0.3 and 0.05 respectively. Experts were then given the opportunity to revise their estimates based on these values (or justify significant differences based on knowledge of other relevant species- or location-specific factors [e.g. emerging threats, life-history or relative protection in reserves]) and were asked for a qualitative assessment of confidence in the final value (*Very confident*, *Confident* or *Not confident*).

### Calculating likelihood of success

For each action at each site within a project, experts were asked to estimate the likelihood of success (0–1) in three different contexts: *Input success* (feasibility) describes the likelihood that managers can successfully proceed with implementing the action, given physical, legal or socio-political constraints (e.g. will poison baiting to control predators be approved, given proximity to residential areas?). *Threat outcome success* describes the likelihood that the action will successfully control the threat in terms of extent or severity (e.g. will poison baiting reduce local predator densities?). *Species outcome success* describes the likelihood that the action will lead to a positive population response (via improving survival and/or reproduction) at the site (e.g. will the local population increase in response to reduced predator densities?).

Likelihood of success scores for each action are assumed to be independent of one another (questions to experts were framed to emphasise this independence; e.g. ‘*assuming* local predator densities *are* reduced by baiting, will the target species’ population increase?’), therefore, the product of all three scores equates to the estimated probability of the action achieving its ultimate objective. Obviously making predictions about the long-term likelihood of success of any action is difficult and prone to uncertainty, especially where there is limited empirical evidence to draw on. Thus, an additional qualitative assessment of expert uncertainty was collected for each estimate (*Very confident*, *Confident* or *Not confident*). A high level of disagreement between experts was interpreted as there being low confidence in the final estimate.

The overall likelihood of success score for a project (*L*) was calculated using:

$$L=\prod_{i=1}^{n}\left(I_{i}T_{i}S_{i}\right)$$

where *I*_*i*_ = input success, *T*_*i*_ = threat outcome success and *S*_*i*_ = species outcome success for the *i*th action, for a project with *n* actions [6].

Where workshop participants did not have experience in the implementation of specific types of management actions (e.g. vertebrate pest control, landholder agreement negotiation and ex-situ flora management), likelihood of success scores were based on estimates from OEH staff with a role in coordinating relevant management themes state-wide.

### Calculating cost

The cost of implementing all management and monitoring actions within a project over a 50 year period were calculated and summed to establish a total project cost. Fifty years was considered sufficient to ensure fair comparisons between projects with different cost profiles (e.g. high initial outlay with low ongoing costs versus moderate and stable annual costs) and short enough to forecast with relative accuracy. Generally it would be more appropriate to estimate costs over an equivalent time horizon to benefits (i.e. 100 years), however, the shorter timeframe was considered more appropriate here, given the greater uncertainty associated with such long-range forecasts and the fact that in pilot analyses, 50-year and 100-year cost estimates were found to be highly correlated.

Costs were estimated by operational experts where appropriate. In addition, a schedule of standard costs associated with common management activities was developed using relevant sources including OEH expenditure tracking databases, land management literature and information held by natural resource management authorities. All human resource costs (e.g. time required for inter-agency and landholder liaison) were also included using applicable agency or contract rates. Estimated costs were applied to all actions as a first estimate; with refinement to account for site-specific variation occurring at the review stage (see below). The full cost of implementing all actions was included in the project total, irrespective of whether or not these costs were already being met by grants or in-kind support (e.g. volunteers) or existing government programs. Costs associated with maintaining existing assets (e.g. fences) were included, but initial build costs were excluded.

Given the long timeframe over which costs are estimated, a discounting factor was used to accurately calculate the net present value of future costs. A static discount rate of 0.01 per year was applied following [6] i.e.:

$$C_{i}=\sum_{t}^{50}\frac{C_{i,t}}{{\left(1+r\right)}^{t}}$$

Where *C* is the cost of project *i* in year *t* and *r* is the discount rate (0.1). This reflects the fact that actions implemented in the future are likely to be cheaper in today’s dollars.

Where two or more projects proposed a similar action at the same location in the same year(s), the costs associated with implementing that action were shared between the relevant projects. If the costs (assumed to correlate with scale/intensity) of the shared action proposed by each project were identical, this amount would be divided equally among the relevant projects. If the costs differed, the highest (assumed enough to meet the objective of all shared projects) was apportioned among each relevant project according to the relative cost of the action proposed by each.

The prioritization algorithm ran iteratively, removing the last-ranked project following each iteration, until the total cost of implementing all remaining projects was less than the stated total budget. Each time a project was removed, any remaining projects that previously shared costs with that project would no longer do so (under the assumption that the removed project would not be implemented in full and therefore not confer a benefit). However, given the difficulty in designating a fixed total budget for SoS (and the high associated uncertainty)–due to there being numerous stakeholders, in-kind contributions and complementary programs–we assumed an excess total available budget, exploiting all opportunities for cost-sharing over 50 years. Given the relatively low frequency of cost-sharing in our data set (species were allocated to the *site-managed* stream based on attributes that reduced the likelihood of co-occurrence; i.e. distributed in small disjunct populations), the final project rankings were less sensitive to this assumption than to other sources of uncertainty (see *Incorporating uncertainty* below).

### Project review

Draft projects were sent out for review to relevant land managers associated with nominated management sites, as well as to groups/individuals with responsibility for, or experience in managing the relevant species or habitat. This included reserve managers, local government and natural resource management agency staff and other state agencies. This provided quality assurance and an opportunity to ground-truth and refine project details, especially with respect to the feasibility of management actions and their associated effort/cost and the scale and intensity of threatening processes on the ground.

Once projects begin to be implemented, ongoing (significant) changes in response to adaptive management or other feedback are made in consultation with species experts and reviewed by a technical reference group composed of several experts for each taxonomic group.

### Project prioritization

The overall objective of the prioritization was to maximize the number of species that could be secured for a given budget. Therefore, (*sensu* [6]) we applied the formula;

$$P=\frac{Benefit\;(B)\times Likelihood\;of\;success\;(L)}{Cost\;(C)}$$

where *P* = priority score, to calculate an index of cost-efficiency for all projects. Projects were then ranked according to *P* to generate an initial priority list for investment.

### Incorporating uncertainty

Uncertainty associated with the elicitation of value estimates and calculation of the parameters *B*, *L* and *C* was likely to come from both incomplete understanding of species’ ecology (structural uncertainty) and the inherent stochasticity in environmental variables over long time periods (unpredictability). To quantify and assess the effects of this uncertainty on *P* and priority rankings we used Monte Carlo simulation models [25]. Likelihood intervals for all values of *B* and *L* were generated by transforming the associated qualitative confidence categories from *Very confident*, *Confident* or *Not Confident* to quantitative intervals ±0.05, ±0.1 and ±0.2 respectively [32]. The intervals for *C* were applied as a constant ±30% based on an analysis of OEH financial planning and expenditure on environmental management activity for the 2011/12 financial year (OEH unpub.). Sites for which no recent (i.e. <5 years) confirmation of on-ground conditions was available were assumed to have greater uncertainty related to cost (i.e. the scale or intensity of threats were more difficult to predict), therefore, for this subset of projects values of *C* were assigned a constant confidence interval of +100%/-30% (to reflect the greater likelihood of unforeseen costs).

For each input parameter in the prioritization equation, Monte Carlo simulations were run sampling from a triangular distribution (there being no theoretical basis for using a normal distribution) centred on the estimate and bounded by the limits of the relevant intervals defined above (*L* truncated with lower limit = 0.1). For each of 10,500 simulations, *P* was calculated for each project and all projects were ranked, generating a frequency distribution of simulated ranks for every project. Using the 2.5 and 97.5 percentile values from these distributions, a 95% likelihood interval for project priority rank was determined.

Finally, the rank likelihood intervals were used to resolve the priority list into five priority bands. All projects with a lower limit ranking in the top 30% (i.e. above 70^th^ percentile) were allocated to Band 1. All projects with their full interval ranking within the next 40% (i.e. 30^th^ to 70^th^) were allocated to Band 3. All projects with an upper limit below the 70^th^ percentile were allocated to Band 5. All projects having likelihood intervals intermediate to Bands 1 and 3 or Bands 3 and 5 were allocated to Bands 2 and 4 respectively. Any project with an interval large enough to span two or more Bands using the above criteria, was allocated to the lowest relevant band.

To assess the relative effect size (sensitivity) of each input parameter in the prioritisation equation on the output (*P*), we ran separate Monte Carlo simulations (10,500) to generate estimates of the expected change in *P* attributable to a 0.1 change in *B* and *L*, and a $1,000,000 change in *C*. These values varied with variation in *P*, therefore estimates were produced for each data point (i.e. 368 species). The three input parameters were also tested for collinearity with (log-transformed) *P* using Pearson’s product-moment coefficient.

The cost-sharing and prioritization analyses were conducted using the Super Region Poly Tool for ArcGIS 9.3 [33] and a custom package (developed by W. P., RM, Belinda Mellish and Liana Joseph; code available at https://github.com/projectprioritisationprotocol/ppp) for R 2.15.1 [34]. For the regression modelling we used the QuantPsyc package for R 2.15.1 and we used J [35] for the Monte Carlo analysis.

## Results

An outline of each of the 368 projects developed, including management site maps and summary information on critical threats and proposed management actions, can be found at http://www.environment.nsw.gov.au/savingourspeciesapp/managementstream.aspx?managementstream=sitemanaged (Note: species projects are dynamic/updateable and may change over time).

In the final priority list, 80, 70, 45, 84 and 89 projects were assigned to priority bands 1, 2, 3, 4 and 5 respectively. Flora were more likely to be represented in higher priority bands, with only 3 fauna in Band 1 (9 would be expected under uniform random assignment to bands). The species list assigned to priority bands can be found at http://www.environment.nsw.gov.au/resources/threatenedspecies/prioritybands.pdf.

Values of *B* ranged from 0.05–0.95 (mean [SD] = 0.54 [0.29]), values of *L* ranged from 1.21x10^-5^–1.00 (0.27 [0.29]), and costs (*C*) ranged from $16,876-$23,462,859 ($1,334,874 [2,489,181]).

The mean likelihood interval for project rank was 60 projects (SD 35; range: 1–174), with intervals more likely to be smaller for very high or very low (i.e. Bands 1 and 5) ranked projects (mean length = 35 [SD 20]) than medium-ranked (i.e. Bands 2–4) projects (mean length = 80 [SD 31]).

**Fig 3:**
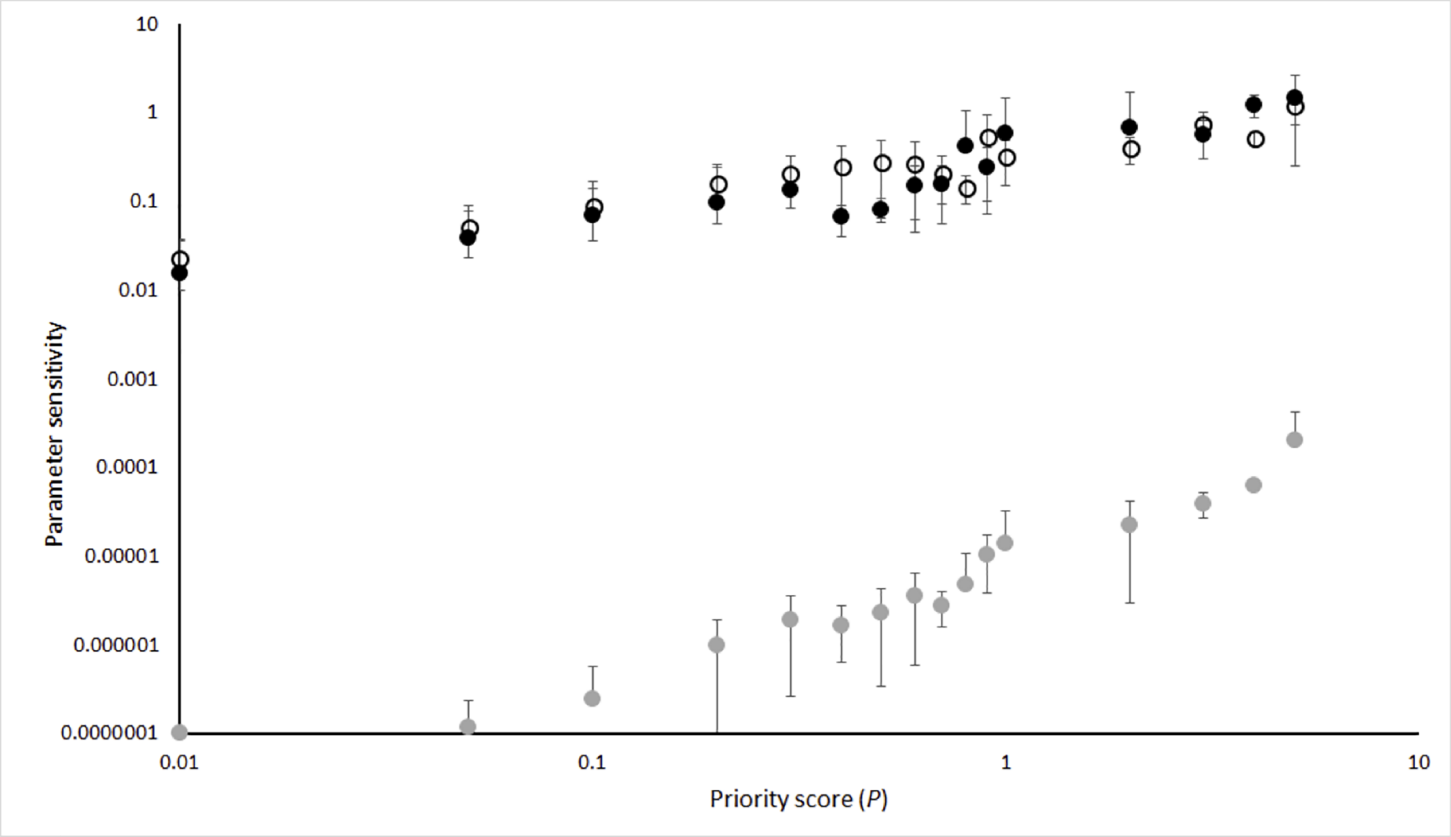
Sensitivity of priority score to change in prioritization parameters. Mean (+/-standard deviation) sensitivity to *Benefit* (solid circles), *Likelihood of success* (empty circles), and *Cost* (grey circles), to in relation to variation in priority score (*P;* categorised into 17 bins, on a log-log scale).

The sensitivity of *P* to changes in *B* (range: 4.56x10^-4^–4.04) was similar to that for *L* (range: 7.37x10^-4^–1.94), which were both, on average, approximately four to five orders of magnitude greater than the sensitivity to changes in *C* (range: 10^−7^–6.52x10^-4^) (Fig 3).

**Fig 4:**
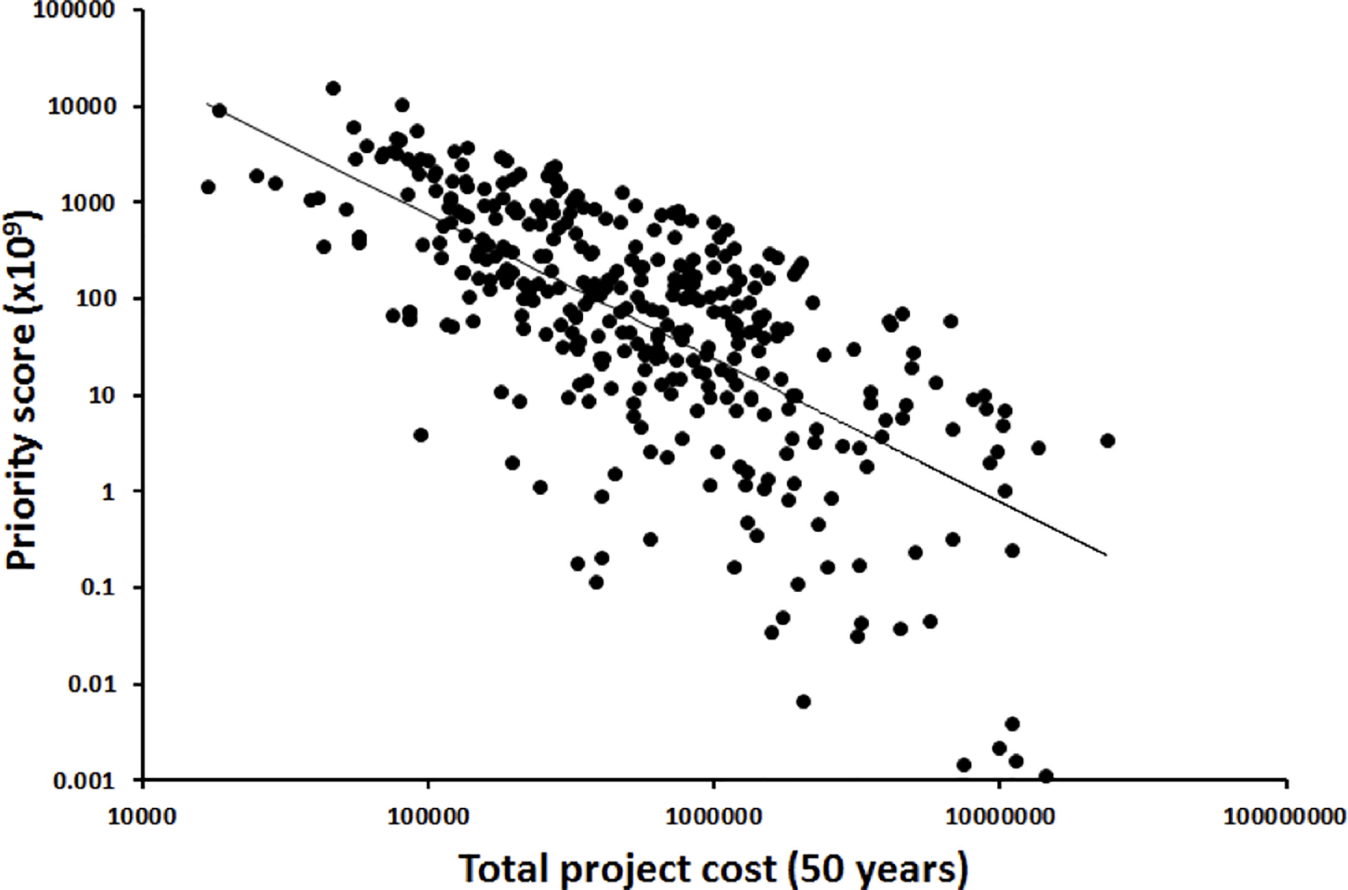
Relationship between project cost and project priority score. Total (50 year) project cost (*C*) (log scale) versus project priority score (*P*) (log scale) for 368 projects (*R*^2^ = -0.69, β = -1.49, *p* < 0.001). Exact values of *C* and *P* are presented (i.e. without incorporating uncertainty).

As expected, there was a significant negative correlation between (log-transformed) *C* and (log-transformed) priority score (*P*) (*R*^2^ = -0.69, β = -1.49, *p* < 0.001; Fig 4). There was no significant relationship with *P* found for either *B* or *L*.

Expert estimates of *P*_*n*_ deviated slightly (although not significantly; *df* = 366, *p* = 0.054) from the anchoring values based on IUCN threat categorisation (mean absolute difference = 0.15 [SD = 0.19], range: 0–0.85).

**Fig 5:**
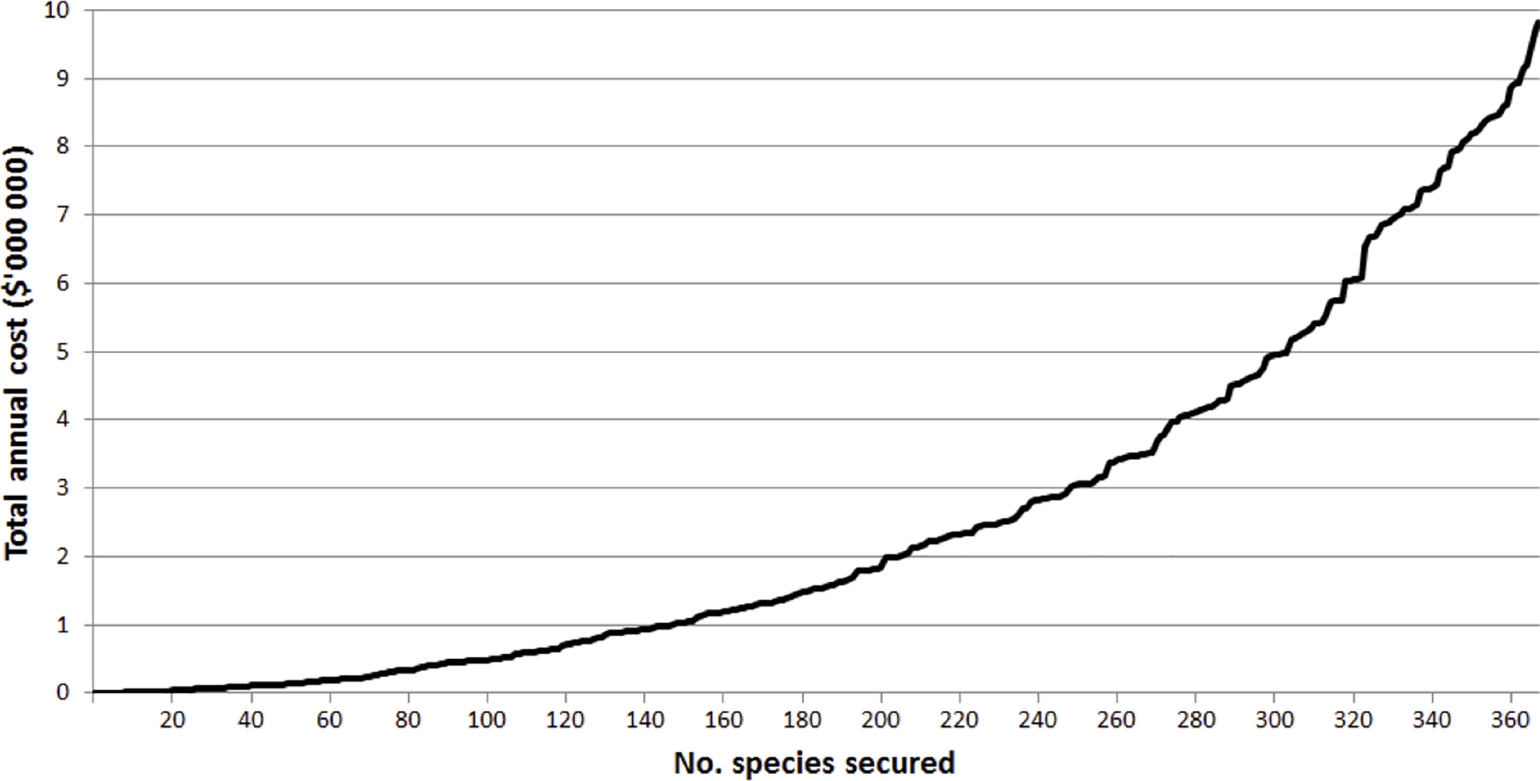
Cumulative estimated total annual cost of securing increasing numbers of species in priority order. 50-year average annual project costs presented, not incorporating uncertainty in *P*.

The total annual implementation cost for all projects (sum of mean annual costs) was $9,824,669. The return on investment curve with respect to number of species secured was exponential, with the first ranked 100 species costing approximately $483,000, the first 200 costing $1.82 million, and the first 300 costing $4.94 million (Fig 5).

## Discussion

By employing an objective and transparent approach to prioritizing investment in threatened species management, we were able to maximise the likelihood of meeting the OEH program objective–securing the greatest number of species for 100 years–with limited resources. This approach, adopted by the SoS program and reported here represents a significant departure from the previous strategy for managing threatened species in NSW (and most other jurisdictions globally). The framework improves outcomes by clearly articulating to all stakeholders what is required, where and when in order to meet specific objectives, within financial and logistical constraints. The planning phase of the program has already been demonstrably more cost-effective than the Recovery Planning process–producing detailed, practical management prescriptions for more than five times the number of species in less than a quarter of the time (and for a fraction of the cost). It has also allowed government to communicate priorities for investing public funds in a transparent, consistent and objective fashion, and to be accountable for those decisions.

In terms of implementation, complete conservation projects (i.e. implementation of all critical actions) for more than 300 (site-managed) species are planned for investment in 2017 thru 2021 under SoS. This compares to 49 species with implementation of all priority actions under the previous program in 2007 thru 2011 (246 species had ≥ 50% of priority actions implemented) ([13]; OEH unpub.). Admittedly, assessing the impact of applying a PPP is difficult, given that it is conflated by a significant increase in investment (the NSW government committed $100 million to SoS 2016–2021 [36]), however, the fact of this unprecedented investment is itself evidence of the political value of adopting an objective, cost-efficiency based approach to prioritisation.

### Dealing with uncertainty

The generally poor availability and reliability of empirical data on the ecology and demography of threatened species is a pervasive constraint on effective decision-making [37–39]. Therefore, the use of expert opinion to supplement available data, as reported here, is relatively widespread but incorporates significant uncertainty [25]. There has been some criticism of quantitative methods for prioritizing investment in threatened species, citing high variability, subjectivity and uncertainty in expert opinion as insurmountable obstacles to producing objective and reliable model outputs [40]. Such criticism is important in highlighting the potential effects that these sources of uncertainty have on the validity and applicability of prioritizations (and the likelihood that projects will meet their objectives), and the importance of how uncertainty is addressed.

Interestingly, project priority was most sensitive to variation (and therefore estimation uncertainty) in benefit and likelihood of success, compared to cost. This result underscores the importance of improving the evidence base for quantifying (and generalising) species’ response to management via adaptive management and long-term monitoring. These data not only improve the precision and reliability of prioritization algorithms by directly informing estimates of benefit and likelihood of success, but can also improve cost-effectiveness indirectly via informing management targets. For example, understanding the relationship between management effort and threat response, and consequent population response, allows for the development of targets for effort, threat and population outcomes that optimize within-project–and therefore overall–cost-effectiveness.

Uncertainty associated with the prioritization was addressed in several ways. Sources of uncertainty were identified at the coarse scale, where species with below a threshold level of data or knowledge available are excluded from the prioritization (allocated to the *data-deficient* management stream) and at the finer scale, where uncertainty in parameter estimates is treated quantitatively (see *Incorporating uncertainty*). In the longer term, input parameters, along with other data on species demography, action implementation and outcomes that are stored in a database (with summary data made available for public review via a web platform) can be continually refined and updated based on new information (e.g. likelihood of success estimates can be revised based on management effectiveness monitoring). Ideally, over time, the database will facilitate reducing uncertainty as empirical data supersedes expert opinion (for example, longitudinal data on population response to management could replace highly uncertain estimates of long-term viability [*P*_*w*_] to inform calculation of *Benefit*). Furthermore, the resolution of the prioritization and the extent to which it drives decision making can both be increased for future iterations in proportion to overall confidence in the input data (e.g. resolution into five broad bands was considered appropriate for the initial dataset, given the relatively low proportion of empirical data compared to expert opinion).

### Facilitating decision making

Using quantitative methods to develop a prioritization of threatened species management projects is only the first step in the decision making process with respect to allocating available resources. Simply starting with the highest ranked project and moving down the list funding all projects until the budget is exhausted, without any other considerations, would likely result in perverse outcomes (e.g. discontinuing projects that have accumulated significant intangible benefits such as community engagement, longitudinal data or institutional capacity). SoS uses the rankings produced by the PPP as one (important) element supporting investment decisions. Other considerations include attributes of projects that relate to benefit, likelihood of success and cost, but are not easily quantifiable in a consistent and defensible way. For example, established community capacity and expertise, efficiencies created by alignment with other government investments, and species’ ecological function (i.e. ‘keystone’ concept).

Furthermore, under SoS the PPP is used as a dynamic, adaptive tool, incorporating changes to projects (including the number and type of management actions and sites, estimates of cost and likelihood of success) on an annual basis. Annual project reprioritization, however, does not necessarily directly inform investment decisions. For example, if changes to a committed project’s *Benefit*, *Likelihood of success or Cost* result in significantly reduced cost-effectiveness and a drop in rank below a particular funding threshold (or below other species without investment), funding for that project is not automatically ceased. Over the long term, the risk of poor outcomes and reduced cost-efficiency is far greater when projects are not given appropriate time to demonstrate a response [41]. Updating the PPP annually can, however, inform investment in new projects with additional/excess budget. This is likely to be required, even under a fixed annual budget allocation, due to the typical profile of project budgets (i.e. relatively high establishment costs in years 1–3 followed by lower ongoing costs) allowing investment in additional projects as the program progresses.

For a large-scale program like SoS, PPP is not the only available method for maximising cost-effectiveness of investment, nor is the particular application of PPP reported here. Prioritization approaches such as priority threat management (e.g. [42], [43]) that have a greater focus on complementarity, may be more appropriate when assets (e.g. species, locations) are large and frequently overlapping in space and management requirements–e.g. *landscape* species under SoS (currently excluded from the PPP). Alternative applications of PPP to that described here may also improve return-on-investment by improving complementarity; in particular, management site could replace species (project) as the unit of prioritization. This would enable comparison amongst and evaluation of the most cost-effective combination of management sites (both within species and overall), potentially identifying a more cost-effective solution than reported here. This approach, however, comes with significant additional challenges: i) it requires the calculation of relative benefit for each site, for which informative data is generally unavailable and expert opinion is highly uncertain. Other authors (e.g. [44]) have simply assumed equivalent contributions of sites to species viability, however, this is obviously a simplification of reality and if adopted may constrain the identification of maximally cost-effective solutions. ii) It is much more computationally expensive–the number of permutations generated by >1000 (irregular shaped) management sites and >100 activity types may be too large for commonly-used conservation planning software.

### Risks of a cost-effectiveness approach

Investing in management at sites representing only a subset of a species’ geographic range inherently increases extinction risk, compared to managing the species everywhere it is known to occur [23–24,45]. This is likely to be true for all species, due to various factors, most of which relate to adaptive capacity and/or resilience to the effects of environmental variability. When determining investment priorities, however, this risk must be weighed against the benefit of securing a larger number of species that is conferred by taking a cost-effective approach. To ensure that the appropriate balance in regard to this trade-off is met, it is important that projects are continually reassessed against their long-term objectives using available data and predictive tools (e.g. population viability analysis, climate change impact modelling).

### Conclusion

The fundamental components of this approach–explicit recognition of the benefit, likelihood of success and cost of different interventions when making investment decisions–are applicable to any natural resource management context. We hope that its application to threatened species in NSW provides a model for other jurisdictions in Australia and beyond. In response to criticisms of PPP or similar cost-efficiency based approaches to investment in threatened species [46], our practical experience reported here demonstrates that the alternative to strategic prioritisation is non-strategic prioritisation. The primary difference is that the former maximises outcomes for threatened species and provides transparency about what those outcomes are, while the latter does neither.

## Supporting information

S1 Dataset BrazillBoastetal_dataset.xls. The spreadsheet contains the data required to calculate priority score for each species included in the prioritization protocol. There are two worksheets:
*PerSpeciesCostBenefit* includes one record per species, with values for total 50-year project cost (net present value, with sharing) (*C*), probability of 100-year viability without management (*P*_*n*_), with management (*P*_*w*_), project benefit (*B*; *P*_*w*_*-P*_*n*_) and uncertainty bounding values (confidence) used in the Monte Carlo analysis (i.e. +/- 0.05, 0.1 or 0.2).
*PerActionLikelihoodOfSuccess* includes one record per management action (many-to-one relationship with projects), with input, output and outcome success and associated uncertainty bounding values (confidence; as above).
(XLS)
